# Effectiveness and Safety of Pharmacological Therapy for Osteoporosis in Frail Elderly Individuals: Systematic Review and Meta‐Analysis

**DOI:** 10.1002/hsr2.73226

**Published:** 2026-10-05

**Authors:** Anna Julia Lacerda Raposo Freire, Fernanda Martins Gazoni, Eduardo de Holanda Carvalho, Fânia Cristina Santos

**Affiliations:** ^1^ Department of Medicina ‐ Geriatrics and Gerontology Discipline Federal University of São Paulo (UNIFESP) São Paulo Brazil

**Keywords:** aged, drug therapy, frailty, osteoporosis, osteoporotic fractures

## Abstract

**Background:**

Frailty and osteoporosis are interrelated conditions that predominantly affect older adults. The greater the individual's frailty, the greater the probability of suffering a fracture and the greater the risk of fractures occurring in the future. Therefore, this study aimed to verify whether anti‐fracture pharmacological therapies in frail individuals with osteoporosis are effective and safe.

**Methods:**

A systematic review of the literature was conducted using randomized clinical trials and observational studies. Participants aged 60 years or older, living in the community or in a long‐term care facility, diagnosed with frailty and osteoporosis and with a history of anti‐fracture medication use were evaluated. A meta‐analysis was performed when appropriate. This review was conducted and reported following the PRISMA 2020 guidelines.

**Results:**

Six studies with 42,851 frail elderly were included. Five were conducted in long‐term care facilities (3 observational and 2 randomized clinical trials) and 1 observational study included community‐dwelling elderly after hospitalization due to fragility fracture. All studies assessed fractures; four evaluated mortality, and one assessed bone mineral density. Three studies compared bisphosphonates to a non‐antiosteoporotic treatment; one compared bisphosphonate, denosumab, and teriparatide to placebo; one compared denosumab and zoledronic acid to teriparatide; and one compared sequential teriparatide with alendronate to alendronate. Meta‐analysis pointed to a potential reduction in hip fracture risk and mortality with bisphosphonates in institutionalized frail elderly. It also demonstrated that bisphosphonates can be safe with limited nonserious side effects.

**Conclusion:**

The findings suggest a possible benefit of pharmacological treatment in reducing hip fractures and mortality in frail elderly. The real impact of the anti‐fracture therapies on those individuals still needs to be better studied.

## Key points

Frail elderly have a higher risk of fracture | Pharmacological therapies may improve bone mineral density and suggest a possible reduction in fracture risk in frail elderly, although current evidence is limited.

Why does this paper matter?

This is the first systematic review and meta‐analysis of anti‐fracture therapies in frail elderly, synthesizing evidence to guide treatment decisions in real‐world geriatric care.

## 1 Introduction

The increased prevalence of age‐related chronic diseases associated with aging represents a growing challenge to public health [1]. Osteoporosis is more common in individuals over 70 years of age since physiological bone remodeling is reduced, resulting in a negative bone balance [2]. It is a systemic skeletal disease, characterized by the reduction of bone mass and degradation of the trabecular structure leading to bone microarchitectural deterioration, increasing bone fragility and risk of fractures [1, 3]. The incidence of hip fractures is 30% and of vertebral fractures is above 40% among women over 80 years [2]. Fragility fractures can increase hospitalization, long‐term care, reduced quality of life, disability and death [1, 3].

Frailty is characterized by a progressive and increasing loss of function and physiological reserve of multiple systems, with vulnerability to stressful events [1, 2, 4]. A complex mechanism of aging, influenced by genetic, epigenetic, and environmental factors, leads to a physical decline affecting several organs and systems [2, 3]. Relevant clinical manifestations include impaired muscle strength and balance, and reduced gait speed [4].

Some authors suggested that osteoporosis and frailty share similar pathophysiological pathways, especially through inflammatory mechanisms [1]. Frailty is strongly associated with reduced bone mineral density, as well as a higher risk of falls and fractures [4, 5]. Its presence can be directly linked to the imminent risk of fracture (probability of a new fracture in the first 12–24 months) [6], and is therefore an independent predictor of osteoporotic fractures. The greater the individual's frailty, the greater the probability of suffering a fracture and the greater the risk of fractures occurring in the future [3]. For instance, the management of osteoporosis should consider not only bone density, but also the individual's degree of frailty, adjusting the choice of antifracture therapy to the level of fracture risk, considering the safety and effectiveness of each medication [4].

Fracture risk assessment is based primarily on bone mineral density and on FRAX (Fracture Risk Assessment Tool) [7, 8], but the absence of frailty as an additional criterion could underestimate the risk of fracture. Bartosch et al. [8] demonstrated that elderly frail women classified as low risk of fracture by FRAX were identified as high risk of hip fracture after also considering frailty. Incorporating this parameter into FRAX would be an option to correct this limitation, however further studies including cost‐effectiveness analyzes should be conducted to assess its applicability in clinical practice. The recognition of frailty as a clinical diagnosis is widely accepted, however the best alternatives for its prevention and treatment are still controversial [9].

There is no standardized frailty scale for classifying the elderly population. Some instruments are widely used to assess frailty in the elderly such as Fried phenotype (the frailty phenotype, Fried et al.) [2, 4, 10], frailty index (FI, Rockwood et al.) [2, 4, 11], and Clinical Frailty Scale (CFS, Rockwood et al.) [12, 13]. The authors of some studies use their own criteria to classify older adults as frail. Hosoi et al. [14] considered frailty to be any condition requiring nursing support or care, and Zullo et al. [15, 16, 17] used tools derived from routinely collected datasets, such as scales generated from the Minimum Data Set (MDS) and the Online Survey Certification and Reporting System (OSCAR), instruments considered pragmatic by the authors to measure frailty [18]. In fact, there are clinical factors associated with frailty in older people living both in long‐term care facilities and community settings and these reinforce the significance of identifying high‐risk individuals and implementing adequate interventions to mitigate frailty, regardless of where they live [19].

The authors of this review believe that treatment for osteoporosis in the elderly should always consider the presence or not of frailty, given the greater risk of adverse outcomes. Furthermore, the choice of medication should be based on its safety and cost‐effectiveness, always considering the presence of multiple comorbidities and polypharmacy, which makes individualization of osteoporosis treatment necessary in frail elderly individuals [2, 4, 5].

The aim of this review was to determine whether pharmacological therapy in frail individuals at high risk of fracture is effective and safe.

## 2 Methods

The protocol was registered with PROSPERO (number CRD42025641121; https://www.crd.york.ac.uk/PROSPERO/view/CRD42025641121). No major amendments to the protocol were made.

### 2.1 Study Selection

Studies were selected involving elderly participants aged 60 years or older, of both sexes, of any nationality, living in the community or in long‐term care facilities, with frailty regardless of the criteria used to classify it, and with a diagnosis of osteoporosis with or without previous fracture, as defined by the World Health Organization (WHO) criteria [20], or with a high risk of fracture classification by FRAX.

Participants with terminal illnesses, or with a life expectancy of less than 1 year or with osteometabolic diseases (such as Paget's disease, osteomalacia, pathological fractures) were excluded.

### 2.2 Search Methods for Identifying Studies

The first search was initially conducted on September 30, 2024, for observational studies or randomized clinical trials in electronic databases, including the Cochrane Central Register of Controlled Trials (CENTRAL), MEDLINE (via PubMed), EMBASE (via Ovid), and LILACS (Latin American and Caribbean Health Sciences Literature). No restrictions on date or language were applied, and review articles were excluded. The search strategy combined controlled vocabulary and free‐text terms related to the following keywords: “aged,” “frailty,” “osteoporosis.” A study published in 2025 was included after the first analysis during the period of extensive searching and reading of literature.

An updated search was conducted on March 3, 2026, for observational studies or randomized clinical trials in electronic databases, such as the Cochrane Central Register of Controlled Trials (CENTRAL), MEDLINE (via PubMed); EMBASE (via Ovid) and LILACS (Latin American and Caribbean Health Science Information database), with no restrictions on date or language, only excluding review articles The update search was revised to enhance sensitivity and reproducibility, incorporating both controlled vocabulary (MeSH/Emtree terms) and free‐text keywords. The following core concepts were included: aged, frailty, osteoporosis. Thus, this search strategy combined controlled vocabulary and free‐text terms related to the following keywords: “aged,” “frailty,” “osteoporosis,” “denosumab,” “teriparatide,” “bisphosphonates,” “alendronate,” “risedronate,” “zoledronic acid,” “romosozumab,” and “abaloparatide” (to methodological transparency, it was included a full reproducible search strategies in the Supporting Information S1).

### 2.3 Study Outcomes

The primary outcomes were the number of incident fractures (vertebral [clinical and radiographic], non‐vertebral and hip) and the percentage change in bone mineral density in the lumbar spine, femoral neck and total femur, measured by dual‐energy densitometry (DXA).

Secondary outcomes were mortality, hospitalization, adverse events to treatment, pain, survival and quality of life.

### 2.4 Data Collection and Analysis

Data were systematically extracted in a predefined and standardized manner according to PRISMA and MOOSE guidelines [21, 22]. Two review authors independently selected the articles that met the inclusion criteria, analyzing their titles and abstracts, and separating those that would be read in full. Then, all the data indicated for the analyzes were extracted independently, and when necessary, a third reviewer was consulted for any discrepancies. The quality of the evidence regarding the magnitude of the effect of each outcome was analyzed using the GRADE instrument [23], with classification as high, moderate, low or very low.

A systematic review and meta‐analysis were conducted with a pre‐defined protocol, ensuring that the results were comprehensive and impartial. For the meta‐analysis, the review authors combined data from observational studies and randomized clinical trials, to increase statistical power, since the number of randomized clinical trials was small [24, 25].

In the comparative analysis of the data, a meta‐analysis of events was performed, comparing two groups of drugs in terms of the proportions of events between them. Since there is a lack of evidence regarding calcitonin effect for osteoporosis, being considered as having no effect or a very weak effect on fracture outcomes, the review authors classified calcitonin as a non‐effect comparator [26].

The effect was expressed as presented by each study: risk ratio (RR), hazard ratio (HR) or odds ratio (OD). The meta‐analysis was presented separating the randomized controlled trial from the observational studies. To avoid any misleading or unscientific unified effect calculation, the diamond representing the overall pooled effect was removed, presenting only the results per study design.

The following software was used for statistical analyzes: R version 4.4.2. Copyright (C) 2024 The R Foundation for Statistical Computing.

### 2.5 Certainty of Evidence

The certainty of evidence was assessed using the Grading of Recommendations Assessment, Development and Evaluation (GRADE) [23] approach and classified as high, moderate, low, or very low.

## 3 Results

### 3.1 Research Results

The initial search (September 30, 2024) identified 673 records, of which 6 articles (5 studies) met the eligibility criteria after duplicate removal; one additional study published thereafter was included. The updated search (March 3, 2026) identified 298 records, but no new eligible studies beyond those already included. Overall, 6 studies (7 articles), 57,924 elderly adults (42,851 frail and 15,073 non‐frail), were included (Figure 1).

**Figure 1:**
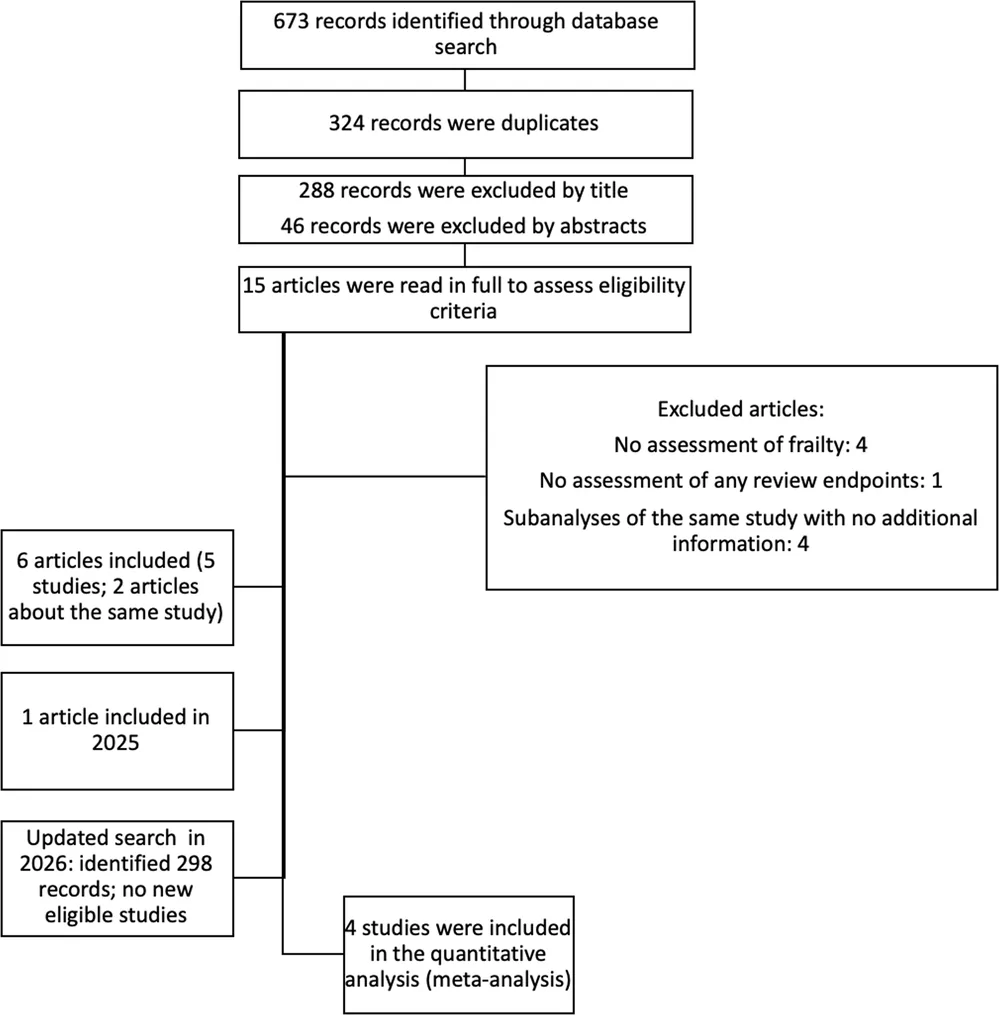
Preferred Reporting Items for Systematic Reviews and Meta‐Analyzes (PRISMA) flow diagram of study selection. A total of 673 records were identified; after removal of duplicates and screening of titles and abstracts, 15 full‐text articles were assessed for eligibility. Seven studies met the inclusion criteria, four of which were included in the quantitative synthesis (meta‐analysis). Updated search in 2026 identified 298 records, with no new eligible studies beyond those already included.

One study compared teriparatide with alendronate or only alendronate; one study, teriparatide with denosumab or zoledronic acid (zoledronate); another compared bisphosphonates, teriparatide, and zoledronate with placebo; and three compared bisphosphonates with a non‐antiosteoporotic treatment (placebo or calcitonin). The characteristics of the included studies are summarized in Supporting Information S1.

Meta‐analyzes were performed only for fractures and mortality, where comparable data were available across studies. Other outcomes, including bone mineral density and comparisons between different pharmacological agents, and adverse events were summarized through narrative synthesis. Hospitalization, pain, survival and quality of life were not reported by the studies.

Four studies analyzed the incidence of hip fractures [15, 16, 17, 27]. A meta‐analysis was performed with two studies comparing data about bisphosphonate and non‐antiosteoporotic treatment [17, 27] (Figure 2).

**Figure 2:**
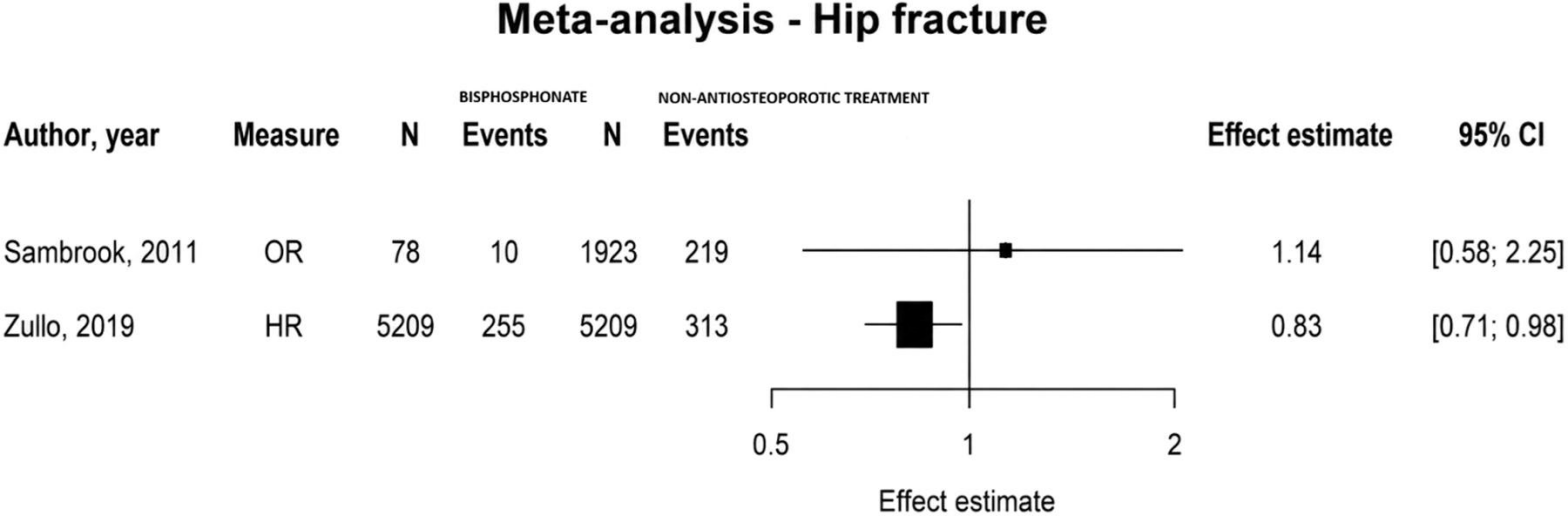
Forest plot of the meta‐analysis of bisphosphonates versus non‐antiosteoporotic treatment for the incidence of hip fracture. The analysis included two studies. N, number of participants; CI, confidence interval. Meta‐analysis – osteoporotic hip fracture.

Sambrook et al. [27] analyzed 78 institutionalized patients in the bisphosphonate group and 1923 in the placebo group (OR = 1.14; 95% CI 0.58–2.25), showing that bisphosphonate group had a 14% higher risk of fracture compared to placebo group, but not statistically significant. The evidence was considered of very low quality due to risk of bias and imprecision.

Zullo et al. [17] analyzed 5209 institutionalized patients in the bisphosphonate group and 5209 in the calcitonin group (HR = 0.83; 95% CI 0.71–0.98), showing that bisphosphonate group had a 14% lower risk of fracture compared to calcitonin group and indicating a statistically significant reduction in the risk of hip fracture associated with bisphosphonate, although the evidence was considered of very low quality due to risk of bias.

Zullo et al. [16] studied bisphosphonate and calcitonin but did not demonstrate the numerical HR value and its confidence interval. They reported a crude rate of 5.4% for the entire cohort, without a breakdown by treatment arm.

Zullo et al. [15] demonstrated that denosumab was more effective in reducing the risk of hip fracture compared to teriparatide in institutionalized elderly individuals (HR = 0.39; 95% CI 0.22–0.7) while zoledronate showed no difference when compared to teriparatide.

Two studies [17, 28] analyzed the incidence of non‐vertebral fracture in institutionalized elderly people comparing bisphosphonate and a non‐antiosteoporotic treatment (Figure 3).

**Figure 3:**
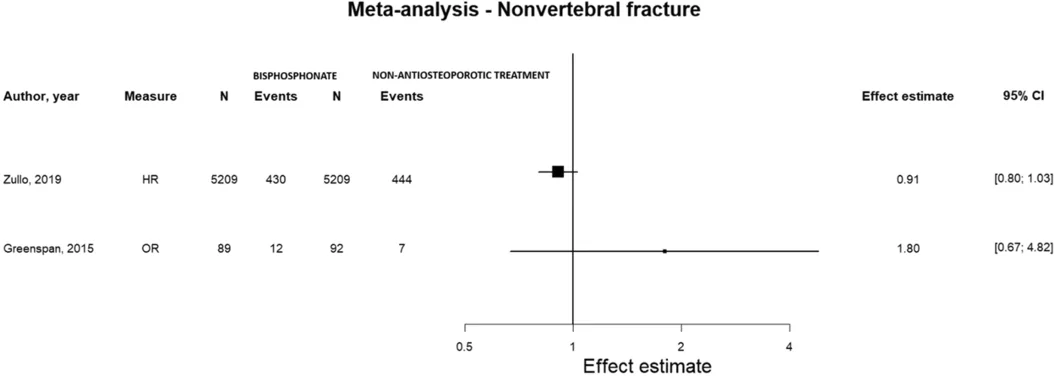
Forest plot of the meta‐analysis of bisphosphonates versus non‐antiosteoporotic treatment for the incidence of non‐vertebral osteoporotic fractures. The analysis included two studies. N, number of participants; CI, confidence interval. Meta‐analysis – non‐vertebral osteoporotic fracture.

Zullo et al. [17] analyzed 5209 institutionalized patients in the bisphosphonate group and 5209 in the calcitonin group (HR = 0.91; 95% CI 0.80–1.03), showing that bisphosphonate group had a 9% lower risk of fracture compared to calcitonin group, but not statistically significant. The evidence was considered of very low quality due to risk of bias and imprecision.

Greenspan et al. [28] analyzed 89 institutionalized patients in the bisphosphonate group and 92 in the placebo group (OR = 1.80; 95% CI 0.67–4.82), showing that bisphosphonate group had 80% higher risk of fracture compared to placebo group, but not statistically significant. The quality of the evidence was considered moderate due to imprecision.

Chattaris et al. [29] studied frail elderly outpatients who have suffered a recent hip fracture and demonstrated that bisphosphonates, denosumab and teriparatide used for less than 90 days increased the risk of fractures (HR = 1.24; 95% CI 1.05–1.56), when compared to placebo, and in non‐frail participants the risk was higher and statistically significant (HR = 1.31; 95% CI 1.04–1.65), while in frail it was of lower significance (HR = 1.17; 95% CI 0.93–1.49); when used for more than 90 days it reduced the risk of fractures by 15% for frail (HR = 0.85; 95% CI 0.65–1.12) and 20% for non‐frail individuals (HR = 0.80; 95% CI 0.61–1.04), without statistical significance [29]. Oral bisphosphonates used for more than 90 days reduced the risk of fractures by 17% for non‐frail (HR = 0.83; 95% CI 0.58–1.19) and 13% for frail individuals (HR = 0.87; 95% CI 0.61–1.26), without statistical significance.

Hosoi et al. [14] studied institutionalized elderly people with primary osteoporosis and high fracture risk considered cognitive frail and physical frail and randomly allocated the two groups in: sequential therapy with once‐weekly teriparatide for 72 weeks followed by once‐weekly alendronate for 48 weeks (TPTD group); or monotherapy with alendronate for 120 weeks (ALN group). The incidence of morphometric vertebral fractures in patients with cognitive frailty was lower in the TPTD group but with no significant difference between the TPTD group and the ALN group (RR = 0.72; 95% CI 0.38–1.34, *p* = 0.30). In patients with physical frailty, the incidence of morphometric vertebral fractures was significantly lower in the TPTD group than in the ALN group (RR = 0.50; 95% CI 0.37–0.68, *p* < 0.01). Analyzes of all fractures and clinical vertebral fractures showed no significant treatment effects (RR = 0.8; 95% CI 0.53–1.21; and RR = 0.84; 95% CI 0.15–4.82).

Greenspan et al. [28] demonstrated after 2 years of a single dose of zoledronate that bisphosphonate group had a 24% lower risk of vertebral fracture and a 30% higher risk of any fracture compared to placebo group (OR = 0.76; 95% CI 0.25–2.28 and OR = 1.30; 95% CI 0.61–2.78), but without statistical significance.

Greenspan et al. [28] was the only study reporting BMD data. They demonstrated that one dose of zoledronate increased significantly the BMD in the lumbar spine (LS) and total hip (TH) after 12 months [LS: zoledronate: 3% (SD: ±0.5%), placebo: 1.1% (SD: ±0.5%); TH: zoledronate: 2.8% (SD: ±0.5%), placebo: − 0.5% (SD: ±0.4%)] and after 24 months [LS: zoledronate: 4.5% (SD: ±0.8%), placebo: 0.7% (SD: ±0.5%); TH: zoledronate: 2.6% (SD: ±0.6%), placebo: −1.5% (SD: ±0.7%)].

Five articles published data about four studies and analyzed death [16, 17, 27, 28, 29]. Two were considered for meta‐analysis [27, 28] (Figure 4).

**Figure 4:**
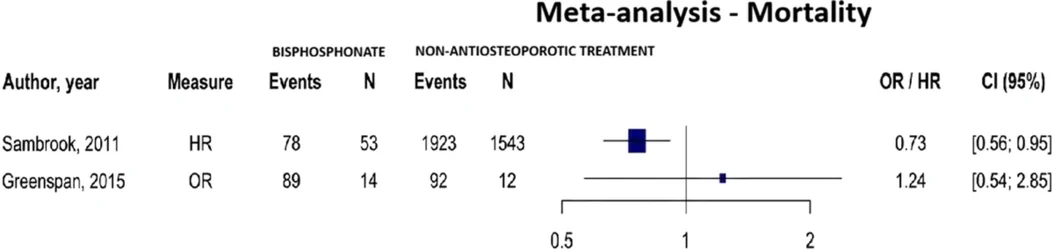
Forest plot of the meta‐analysis of bisphosphonates versus non‐antiosteoporotic treatment on all‐cause mortality. The analysis included two studies. CI, confidence interval; N, number of participants; HR, hazard ratio; OR, Odds ratio. Meta‐analysis – mortality.

Sambrook et al. [27] analyzed 78 institutionalized patients in the bisphosphonate group and 1923 in the placebo group (HR = 0.73; 95% CI 0.56–0.95), showing that bisphosphonate group had a 27% lower risk of death compared to placebo group, indicating a statistically significant reduction in the risk of death associated with bisphosphonate, although the evidence was considered of very low quality due to risk of bias.

Greenspan et al. [28] analyzed 89 institutionalized patients in the bisphosphonate group and 92 in the placebo group (OR = 1.24; 95% CI 0.54–2.85), showing that bisphosphonate group had a 24% higher risk of death compared to placebo group, but not statistically significant. The quality of the evidence was considered moderate due to imprecision.

Zullo et al. [17] presented only descriptive data, reporting 8,189 deaths in the total matched cohort (78.6%), with 77.3% among bisphosphonate users and 80.0% among calcitonin users, without an associated confidence interval, *p*‐value, or statistical model.

Zullo et al. [16] reported only a death rate of 79.0%, also referring to the entire cohort, without indicating which treatment arm it represents or providing any associated measure of effect.

Chattaris et al. [29] reported that using bisphosphonates, denosumab and teriparatide after a fragility fracture, for more than 90 days, reduced mortality in both frail (HR = 0.74; 95% CI 0.6–0.91) and non‐frail individuals (HR = 0.68; 95% CI 0.52–0.89), with statistical significance.

Regarding safety, Zullo et al. [16, 17] did not describe the effect of bisphosphonates on esophagitis and heart failure [17]. Greenspan et al. [28] observed that 97% of participants had at least one adverse event with zoledronate, 64% of which were serious, with no significant difference between the groups. After 3 days following the infusion, the zoledronate group presented more acute symptoms, but with no differences in laboratory safety parameters and in the incidence of cardiac disorders.

Chattaris et al. [29] found no hospitalization difference between frail and non‐frail groups, but no additional data were described. Quality of life or pain were not reported in any of the included studies.

### 3.2 Risk of Bias in the Included Studies

The ROBINS‐I tool [30, 31] was used to assess the risk of bias in prospective and retrospective cohorts (Supporting Information Table S2), and the Cochrane Risk of Bias 2 (RoB2) tool [32] was used for randomized controlled trials (Supporting Information Table S3).

## 4 Discussion

### 4.1 Summary of Primary Findings

As far as we know, this is the first systematic review and meta‐analysis on anti‐fracture therapy in the frail elderly population. Six studies (in seven articles) were included, totaling 42,851 frail elderly, 27,506 of whom were in long‐term care facilities. This review indicated that anti‐fracture therapy with bisphosphonates might have a reduction in the risk of hip fracture and mortality in institutionalized frail elderly according to large observational studies, and it was considered safe, although the results did not reach statistical significance.

All studies included in the review evaluated bisphosphonates and three of them also other agents, including denosumab and teriparatide. Fracture data were analyzed in all studies, four studies addressed mortality, two adverse events and one BMD. None evaluated hospitalization, pain, survival or quality of life. Furthermore, no study has reported data on romosozumab, which is considered as one of the most potent medications to prevent osteoporotic fracture, which would be interesting, considering the vulnerable ones [33].

Very low quality of evidence was observed for the outcomes. The reduction in the quality of evidence has been primarily due to risk of bias (observational studies) and imprecision As shown in Table S1 (Supporting Information), the studies considered the population frail by different instruments. Frailty was also established by scale derived from collected datasets (generated from the Minimum Data Set – MDS) and the Online Survey Certification and Reporting System (OSCAR), which are pragmatic measures of frailty in nursing home (variables derived frailty indices based on the accumulated deficits model) [18]. This could have influenced the quality of the evidence in the present review.

### 4.2 Comparison With Existing Literature

Frailty is a critical global health challenge among older adults. Recent findings showed higher frailty prevalence in nursing homes compared to communities (nursing homes 10%–80.1% and in community settings 3.8%–70.6%.) [19]. Despite this, frail elderly people are excluded from most pivotal osteoporosis trials.

According to some published data, about 85% of frail elderly people have osteoporosis [34] with a risk for any fracture 8 to 9 times greater than that observed among robust elderly individuals [28] and a 36% mortality rate after a hip fracture [35]. However, anti‐fracture therapy remains largely under prescribed in this population [28]. Due to a low number of studies evaluating medications for osteoporosis in frail elderly, there is a lack of data to support therapeutic decision‐making in this group.

Hosoi et al. [14] suggest that assessing frailty in older adults plays a crucial role in managing osteoporosis and making informed decisions about fracture prevention. They analyzed the effectiveness and safety of teriparatide and alendronate in elderly and frail adults and provided insights that may guide treatment strategies in clinical practice. In physically frail women, teriparatide reduced vertebral fractures significantly more than alendronate; however, with little difference in efficacy.

Greenspan et al. [28] evaluated therapy with zoledronate in institutionalized elderly individuals considered frail and found a significant increase in BMD; however, no statistically significant difference for fractures was observed. A post hoc secondary analysis demonstrated a statistically significant increase in BMD at the hip and femoral neck, as well as reduction in bone turnover markers, among frail individuals with cognitive impairment, and similar results in cognitively preserved individuals [36]. Studies in non‐frail elderly have shown that small increases in BMD are associated with substantial reductions in fracture risk, mainly vertebral and hip fractures [37]. The same effect could be suggested for frail elderly individuals, therefore an increase in BMD should also reduce the number of fractures [38].

Two recent studies support the effectiveness of zoledronic acid in older adults, including those with frailty [39, 40]. Ozcan et al. [39] evaluated 156 elderly outpatients with osteoporosis, including frail individuals (23.1%), and found that a single dose of zoledronic acid was effective in 70.5% of participants over a mean follow‐up of 14.4 months, supporting its efficacy even in the presence of multimorbidity and frailty. Similarly, Haeri et al. [40] investigated elderly people from long‐term care facilities, including frail (28.2%) and pre‐frail (41%), and demonstrated that a single infusion of zoledronic acid, following denosumab discontinuation, maintained BMD across multiple skeletal sites and improved bone microarchitecture, according to trabecular bone score.

Nevertheless, our data may demonstrate a potential benefit for treating osteoporosis in frail elderly, as already proven in different studies with non‐frails, in which bisphosphonates, denosumab, and teriparatide have been proven effective in reducing vertebral, non‐vertebral and hip fractures, with statistically significant results [41, 42, 43]. In addition, bisphosphonates are considered safe, as established in literature [44, 45]. Studies in non‐frail demonstrate a protective effect [46, 47, 48], but survival data need to be further evaluated in studies that rule out confounding factors for mortality in frail elderly.

### 4.3 Clinical Implications Specific to Frailty

The lack of a statistically significant reduction in fractures, despite an increase in BMD observed in the study by Greenspan et al. [28], suggests that other factors, such as falls, also play an important role in the fracture risk among frail elderly. It is possible that frail elderly present greater impairment of skeletal integrity, including damage to bone microarchitecture and a limited response to less potent therapies, such as oral bisphosphonates [28]. Therefore, the use of more potent medications, such as denosumab, teriparatide, and romosozumab should be considered, as these agents are associated with significant short‐term reductions in fracture risk [49].

It is important to highlight the risk of esophagitis with oral bisphosphonates, which may be particularly relevant in frail elderly with swallowing difficulties [44].

There was no difference in hospitalization between frail and non‐frail patients who suffered a hip fracture in the study by Chattaris et al. [29]. These findings suggest that, despite the potential benefits in fracture prevention, the impact of bisphosphonates on reducing new hospitalizations still requires further investigation.

This systematic review and metanalyses can contribute to a better understanding of the effects of anti‐fracture medications in frail elderly, in the community or long‐term care facilities. This review suggests that there is a potential benefit of pharmacological treatment in preventing fractures and reducing mortality in frail elderly adults, with acceptable security, but with limited data and a lack of statistical power.

### 4.4 Limitations

The present sytudy has some limitations. The meta‐analysis included randomized clinical trials (RCTs) and observational studies. Analyzing RCTs in conjunction with observational studies presents significant methodological challenges, primarily due to inherent differences in study design, bias structures, and population characteristics, while combining them can provide a more comprehensive view of real‐world effectiveness. Although, analyzing data from different study designs may be possible when there is a need to increase statistical power or when the number of randomized clinical trials is small in the review [24, 25]. The meta‐analysis was limited by the small number of eligible studies and by the use of different effect metrics (HR and OR). However, a structured subgroup analysis was performed to ensure analytical transparency. The observational studies included in the review did not analyze bisphosphonates by type, which prevented the assessment of specific anti‐fracture effect of each medication, even for institutionalized frail elderly people.

Furthermore, the studies analyzed used different classifications for fragility, which may have reduced the statistical strength of the review. The fragility status is currently understood as a multidimensional construct, encompassing clinical, functional, and biological domains, and different instruments may capture distinct aspects of this syndrome, including physical performance measures (e.g., gait speed, grip strength), imaging‐based markers (e.g., muscle mass), and laboratory biomarkers reflecting underlying physiological processes [50]. Three studies with institutionalized elderly [15, 16, 17] used scales that are not usually used to fragility like that one derived from collected datasets (generated from the Minimum Data Set (MDS) and the Online Survey Certification and Reporting System (OSCAR), despite being pragmatic measures of frailty in nursing home residents for standardized characterization of vulnerability within that setting [18].

Another limitation was the use of calcitonin as a “non‐antiosteoporotic treatment”. The authors considered calcitonin as having a placebo‐like effect due to a lack of evidence regarding a plausible dose‐response relationship for calcitonin, and inconsistent evidence suggesting that it has no effect or a very weak effect on fracture outcomes, as previously described in a study [26].

## Author Contributions

Anna Julia Lacerda Raposo Freire contributed to conceptualization, investigation and writing the original draft. Fernanda Martins Gazoni contributed to methodology, data analysis, writing and editing the original draft. Eduardo de Holanda Carvalho contributed to investigation, data curation, and reviewing the draft article. Fânia Cristina Santos was the senior investigator and contributed to conceptualization, methodology, writing the original draft, visualization and project administration. All authors reviewed and approved the final version, and no other person made a substantial contribution to the article.

## Funding

The authors have nothing to report.

## Ethics Statement

This is a review article based on published literature and with no need for ethical approvals and informed consent since it does not involve humans and animals. The authors declare that this manuscript is related to the original work and has not been submitted into other journals. The authors have no ethical issues to declare.

## Conflicts of Interest

The authors declare no conflicts of interest.

## Transparency Statement

The lead/corresponding author (Anna Julia Lacerda Raposo Freire) affirms that this article is an honest, accurate, and transparent account of the study being reported; that no important aspects of the study have been omitted; and that any discrepancies from the study as planned (and, if relevant, registered) have been explained.

## Supporting information


Supporting File

## Data Availability

This study is based on previously published data. All data extracted and analyzed are available in the included articles cited in the article.

## References

[1] Y. Hu , H. Xu , W. Ji , et al., “Prevalence of Frailty in Senile Osteoporosis: A Systematic Review and Meta‐Analysis,” Archives of Gerontology and Geriatrics 130 (2025): 105718, 10.1016/j.archger.2024.105718. 39671885

[2] E. A. Greco , P. Pietschmann , and S. Migliaccio , “Osteoporosis and Sarcopenia Increase Frailty Syndrome in the Elderly,” Frontiers in Endocrinology 10 (2019): 255, 10.3389/fendo.2019.00255. PMC6491670 31068903

[3] G. Li , L. Thabane , A. Papaioannou , G. Ioannidis , M. A. H. Levine , and J. D. Adachi , “An Overview of Osteoporosis and Frailty in the Elderly,” BMC Musculoskeletal Disorders 18 (2017): 46, 10.1186/s12891-017-1403-x. PMC5270357 28125982

[4] J. Lee , J. Kim , C. Jeong , J. Ha , Y. Lim , and K. H. Baek , “Predicting Fragility Fractures Based on Frailty and Bone Mineral Density Among Rural Community‐Dwelling Older Adults,” European Journal of Endocrinology 191 (2024): 75–86, 10.1093/ejendo/lvae080. 38970525

[5] L. K. Liu , W. J. Lee , L. Y. Chen , et al., “Association Between Frailty, Osteoporosis, Falls and Hip Fractures Among Community‐Dwelling People Aged 50 Years and Older in Taiwan: Results From I‐Lan Longitudinal Aging Study,” PLoS One 10 (2015): e0136968, 10.1371/journal.pone.0136968. PMC4562637 26348034

[6] P. Bartosch , L. Malmgren , J. Kristensson , F. E. Mcguigan , and K. E. Akesson , “In Community‐Dwelling Women Frailty Is Associated With Imminent Risk of Osteoporotic Fractures,” Osteoporosis International 32 (2021): 1735–1744, 10.1007/s00198-021-05886-7. PMC8387253 33661308

[7] J. A. Kanis , O. Johnell , A. Oden , H. Johansson , and E. McCloskey , “FRAX and the Assessment of Fracture Probability in Men and Women From the UK,” Osteoporosis International 19 (2008): 385–397, 10.1007/s00198-007-0543-5. PMC2267485 18292978

[8] P. Bartosch and L. Malmgren , “Can Frailty in Conjunction With FRAX Identify Additional Women at Risk of Fracture? A Longitudinal Cohort Study of Community Dwelling Older Women,” BMC Geriatrics 22 (2022): 951, 10.1186/s12877-022-03639-7. PMC9733205 36494774

[9] F. Buckinx , J. Y. Reginster , S. Gillain , J. Petermans , T. Brunois , and O. Bruyère , “Prevalence of Frailty in Nursing Home Residents According to Various Diagnostic Tools,” Journal of Frailty and Aging 6 (2017): 122–128, 10.14283/jfa.2017.20. 28721427

[10] L. P. Fried , C. M. Tangen , J. Walston , et al., “Frailty in Older Adults: Evidence for a Phenotype,” Journals of Gerontology Series A: Biological Sciences and Medical Sciences 56 (2001): M146–M157, 10.1093/gerona/56.3.m146. 11253156

[11] K. Rockwood and A. Mitnitski , “Frailty in Relation to the Accumulation of Deficits,” Journals of Gerontology Series A: Biological Sciences and Medical Sciences 62 (2007): 722–727, 10.1093/gerona/62.7.722. 17634318

[12] K. Rockwood , X. Song , C. Macknight , et al., “A Global Clinical Measure of Fitness and Frailty in Elderly People,” CMAJ: Canadian Medical Association Journal 173 (2005): 489–495, 10.1503/cmaj.050051. PMC1188185 16129869

[13] S. Church , E. Rogers , K. Rockwood , and O. Theou , “A Scoping Review of the Clinical Frailty Scale,” BMC Geriatrics 20 (2020): 393, 10.1186/s12877-020-01801-7. PMC7540438 33028215

[14] T. Hosoi , M. Yunoki , S. Tanaka , et al., “Evaluation of the Fracture Prevention Effects of Teriparatide and Alendronate in Patients With Frailty: A Sub‐Analysis of the Japanese Osteoporosis Intervention Trial‐05,” Journal of Bone and Mineral Metabolism 43 (2025): 448–457, 10.1007/s00774-025-01610-1. PMC12279556 40425867

[15] A. R. Zullo , Y. Lee , C. Lary , L. A. Daiello , D. P. Kiel , and S. D. Berry , “Comparative Effectiveness of Denosumab, Teriparatide, and Zoledronic Acid Among Frail Older Adults: A Retrospective Cohort Study,” Osteoporosis International 32 (2021): 565–573, 10.1007/s00198-020-05732-2. PMC7933063 33411003

[16] A. R. Zullo , M. N. Sorial , Y. Lee , C. W. Lary , D. P. Kiel , and S. D. Berry , “Predictors of Hip Fracture Despite Treatment With Bisphosphonates Among Frail Older Adults,” Journal of the American Geriatrics Society 68 (2020): 256–260, 10.1111/jgs.16176. PMC7002229 31580488

[17] A. R. Zullo , T. Zhang , Y. Lee , et al., “Effect of Bisphosphonates on Fracture Outcomes Among Frail Older Adults,” Journal of the American Geriatrics Society 67 (2019): 768–776, 10.1111/jgs.15725. PMC6705123 30575958

[18] H. Luo , T. Lum , G. Wong , J. Kwan , and J. Tang , “Predicting Adverse Health Outcomes in Nursing Home: A Frailty Measure Using Minimum Data Set 2.0,” supplement, Innovation in Aging 1, no. Suppl1 (2017): 672–673, 10.1093/geroni/igx004.2392.

[19] J. Liu , Y. Zhu , J. K. Tan , A. H. Ismail , R. Ibrahim , and N. H. Hassan , “Factors Associated With Frailty in Older Adults in Community and Nursing Home Settings: A Systematic Review With a Meta‐Analysis,” Journal of Clinical Medicine 13 (2024): 2382, 10.3390/jcm13082382. PMC11050860 38673654

[20] J. A. Kanis , *Assessment of Osteoporosis at the Primary Health Care Level* (WHO Collaborating Centre for Metabolic Bone Diseases, University of Sheffield, 2008).

[21] M. J. Page , J. E. McKenzie , P. M. Bossuyt , et al., “The PRISMA 2020 Statement: An Updated Guideline for Reporting Systematic Reviews,” BMJ 372 (2021): n71, 10.1136/bmj.n71. PMC8005924 33782057

[22] D. F. Stroup , “Meta‐Analysis of Observational Studies in Epidemiology: A Proposal for Reporting,” Journal of the American Medical Association 283 (2000): 2008–2012, 10.1001/jama.283.15.2008. 10789670

[23] G. H. Guyatt , A. D. Oxman , G. E. Vist , et al., “GRADE: An Emerging Consensus on Rating Quality of Evidence and Strength of Recommendations,” BMJ 336 (2008): 924–926, 10.1136/bmj.39489.470347.AD. PMC2335261 18436948

[24] P. E. Verde , “A Bias‐Corrected Meta‐Analysis Model for Combining Studies of Different Types and Quality,” Biometrical Journal 63, no. 2 (2021): 406–422, 10.1002/bimj.201900376. 32996196

[25] S. Metelli and A. Chaimani , “Challenges in Meta‐Analyses With Observational Studies,” Evidence Based Mental Health 23, no. 2 (2020): 83–87, 10.1136/ebmental-2019-300129. PMC10231593 32139442

[26] C. H. Chesnut , S. Silverman , K. Andriano , et al., “A Randomized Trial of Nasal Spray Salmon Calcitonin in Postmenopausal Women With Established Osteoporosis: The Prevent Recurrence of Osteoporotic Fractures Study,” American Journal of Medicine 109, no. 4 (2000): 267–276, 10.1016/S0002-9343(00)00490-3. 10996576

[27] P. N. Sambrook , I. D. Cameron , J. S. Chen , et al., “Oral Bisphosphonates Are Associated With Reduced Mortality in Frail Older People: A Prospective Five‐Year Study,” Osteoporosis International 22, no. 9 (2011): 2551–2556, 10.1007/s00198-010-1444-6. 20959963

[28] S. L. Greenspan , S. Perera , M. A. Ferchak , D. A. Nace , and N. M. Resnick , “Efficacy and Safety of Single‐Dose Zoledronic Acid for Osteoporosis in Frail Elderly Women: A Randomized Clinical Trial,” JAMA Internal Medicine 175, no. 6 (2015): 913–921, 10.1001/jamainternmed.2015.0747. PMC4843134 25867538

[29] T. Chattaris , G. Oh , N. A. Gouskova , D. H. Kim , D. P. Kiel , and S. D. Berry , “Osteoporosis Medications Prevent Subsequent Fracture in Frail Older Adults,” Journal of Bone and Mineral Research 37, no. 11 (2022): 2103–2111, 10.1002/jbmr.4693. PMC9712267 36168189

[30] J. A. Sterne , M. A. Hernán , B. C. Reeves , et al., “Robins‐I: A Tool for Assessing Risk of Bias in Non‐Randomised Studies of Interventions,” BMJ 355 (2016): i4919, 10.1136/bmj.i4919. PMC5062054 27733354

[31] J. A. C. Sterne and J. P. T. R. O. B. I. N. S.‐I. Higgins , “V2: Tool for Assessing Risk of Bias in Non‐Randomised Studies of Interventions [Internet],” Risk of Bias Tools (2024): Nov., https://www.riskofbias.info/welcome/robins-i-v2.

[32] J. A. C. Sterne , J. Savović , M. J. Page , et al., “RoB 2: A Revised Tool for Assessing Risk of Bias in Randomised Trials,” BMJ 366 (2019): l4898, 10.1136/bmj.l4898. 31462531

[33] L. Liu , R. J. Clifton‐Bligh , C. M. Girgis , and M. L. Gild , “Extending the Therapeutic Potential: Romosozumab in Osteoporosis Management,” Journal of the Endocrine Society 8, no. 11 (2024): bvae160, 10.1210/jendso/bvae160. PMC11443337 39355688

[34] J. M. Chandler , “Low Bone Mineral Density and Risk of Fracture in White Female Nursing Home Residents,” Journal of the American Medical Association 284, no. 8 (2000): 972–977, 10.1001/jama.284.8.972. 10944642

[35] M. D. Neuman , J. H. Silber , J. S. Magaziner , M. A. Passarella , S. Mehta , and R. M. Werner , “Survival and Functional Outcomes After Hip Fracture Among Nursing Home Residents,” JAMA Internal Medicine 174, no. 8 (2014): 1273–1280, 10.1001/jamainternmed.2014.2362. PMC4122620 25055155

[36] B. M. Churilla , S. Perera , S. L. Greenspan , N. M. Resnick , and M. P. Kotlarczyk , “Zoledronic Acid and Bone Health in Older Adults With Cognitive Impairment,” Osteoporosis International 33, no. 1 (2022): 293–298, 10.1007/s00198-021-06063-6. PMC8758516 34341833

[37] T. S. Tran , T. P. Ho‐Le , D. Bliuc , J. R. Center , R. D. Blank , and T. V. Nguyen , “Prevention of Hip Fractures: Trade‐Off Between Minor Benefits to Individuals and Large Benefits to the Community,” Journal of Bone and Mineral Research 38, no. 11 (2023): 1594–1602, 10.1002/jbmr.4907. 37638688

[38] M. L. Bouxsein , R. Eastell , L. Y. Lui , et al., “Change in Bone Density and Reduction in Fracture Risk: A Meta‐Regression of Published Trials,” Journal of Bone and Mineral Research 34, no. 4 (2019): 632–642, 10.1002/jbmr.3641. 30674078

[39] B. Ozcan , S. Ceylan , M. Ozcan , et al., “Understanding the Impact of Multimorbidity, Medications and Frailty on Zoledronic Acid Efficacy in Treating Osteoporosis Among Older Adults,” British Journal of Clinical Pharmacology 91, no. 12 (2025): 3511–3520, 10.1002/bcp.70243. 40835589

[40] N. S. Haeri , S. Perera , and S. L. Greenspan , “Impact of Zoledronic Acid on Bone Mineral Density and Trabecular Score Following Denosumab Discontinuation in Older Adults in Long‐Term Care,” Calcified Tissue International 116, no. 1 (2025): 55, 10.1007/s00223-025-01364-y. PMC12280700 40146262

[41] I. R. Reid and E. O. Billington , “Drug Therapy for Osteoporosis in Older Adults,” Lancet 399, no. 10329 (2022): 1080–1092, 10.1016/S0140-6736(21)02646-5. 35279261

[42] M. N. Händel , I. Cardoso , C. von Bülow , et al., “Fracture Risk Reduction and Safety by Osteoporosis Treatment Compared With Placebo or Active Comparator in Postmenopausal Women: Systematic Review, Network Meta‐Analysis, and Meta‐Regression Analysis of Randomised Clinical Trials,” BMJ 381 (2023): e068033, 10.1136/bmj-2021-068033. PMC10152340 37130601

[43] P. Barrionuevo , E. Kapoor , N. Asi , et al., “Efficacy of Pharmacological Therapies for the Prevention of Fractures in Postmenopausal Women: A Network Meta‐Analysis,” Journal of Clinical Endocrinology and Metabolism 104, no. 5 (2019): 1623–1630, 10.1210/jc.2019-00192. 30907957

[44] A. Qaseem , L. A. Hicks , I. Etxeandia‐Ikobaltzeta , T. Shamliyan , and T. G. Cooney , “Pharmacologic Treatment of Primary Osteoporosis or Low Bone Mass to Prevent Fractures in Adults: A Living Clinical Guideline From the American College of Physicians,” Annals of Internal Medicine 176, no. 2 (2023): 224–238, 10.7326/M22-1034. PMC10885682 36592456

[45] C. Delli Poggi , M. Fusaro , M. C. Mereu , M. L. Brandi , and L. Cianferotti , “Cardiovascular Safety and Effectiveness of Bisphosphonates: From Intervention Trials to Real‐Life Data,” Nutrients 14, no. 12 (2022): 2369, 10.3390/nu14122369. PMC9227734 35745099

[46] C. H. Wu , C. C. Li , Y. H. Hsu , F. W. Liang , Y. F. Chang , and J. S. Hwang , “Comparisons Between Different Antiosteoporosis Medications on Postfracture Mortality: A Population‐Based Study,” Journal of Clinical Endocrinology and Metabolism 108, no. 4 (2023): 827–833, 10.1210/clinem/dgac636. 36317591

[47] T. W. Tai , J. S. Hwang , C. C. Li , J. C. Hsu , C. W. Chang , and C. H. Wu , “The Impact of Various Anti‐Osteoporosis Drugs on All‐Cause Mortality After Hip Fractures: A Nationwide Population Study,” Journal of Bone and Mineral Research 37, no. 8 (2022): 1520–1526, 10.1002/jbmr.4627. 35689432

[48] Z. Lan , X. Lin , D. Xue , Y. Yang , M. Saad , and Q. Jin , “Can Bisphosphonate Therapy Reduce Overall Mortality in Patients With Osteoporosis? A Meta‐Analysis of Randomized Controlled Trials,” Clinical Orthopaedics and Related Research 483, no. 1 (2025): 91–101, 10.1097/CORR.0000000000003204. PMC11658732 39172899

[49] K. Ebina , Y. Etani , T. Noguchi , K. Nakata , and S. Okada , “Clinical Effects of Teriparatide, Abaloparatide, and Romosozumab in Postmenopausal Osteoporosis,” Journal of Bone and Mineral Metabolism 43, no. 1 (2025): 3–9, 10.1007/s00774-024-01536-0. PMC11954689 39009890

[50] I. Omarova , A. Yeshmanova , G. Gabdulina , et al., “Challenges in Identifying Biomarkers of Frailty Syndrome: A Systematic Review,” Medicina 61, no. 7 (2025): 1309, 10.3390/medicina61071309. PMC12298523 40731939

