# Supplementary material for: Effectiveness and Safety of Pharmacological Therapy for Osteoporosis in Frail Elderly Individuals: Systematic Review and Meta‐Analysis
Source: Health Sci Rep. 2026 Oct 5;9(10):e73226. doi: 10.1002/hsr2.73226 (PMC13635978; doi:10.1002/hsr2.73226)
Supplement: Supplementary file 1 [file HSR2-9-e73226-s001.docx]

**SUPPLEMENTARY MATERIAL**

**Table S1.** Characteristics of the studies

| **Study** | **Design** | **Inclusion criteria / Study location** | **Duration** | **Fragility indicators** | **Population** | **Intervention** | **Outcomes** |
| --- | --- | --- | --- | --- | --- | --- | --- |
| **Hosoi**  **et al, 2025 [14]** | Randomized, controlled, open-label, clinical trial | Institutionalized, ≥75 years, with osteoporosis/  Japan | 6 years  Mean 2.0 | Any condition requiring nursing support or care (physical fragility) | NT: 718  NW: 718 (100%)  NF: 204  NI: 109  Mean age: 82.3 years (± 4.7) | **Intervention:**  **- Teriparatide and alendronate**  Comparator:  **alendronate** | Primary: morphometric vertebral fractures  Secondary: clinical vertebral fractures, progression of vertebral fractures, nonvertebral fractures and all fractures |
| **)Zullo et al, 2021 [15]** | Observational study (retrospective cohort) | Institutionalized,  >65 years, with osteoporosis /  United States | 5 years  Mean 1.5 | Scale generated in the Minimum Data Set (version 2.0), Online Survey Certification and Reporting System (variables derived frailty indices based on the accumulated deficits model) | NT: 2,019  NW: 1,814 (90%).  NI: 1,441  Mean age: 85.3 years (± 7.7) | **Intervention:**  **- Denosumab**  **- Zoledronic acid**  **Comparator: teriparatide** | Primary: hip fractures ^1^  Secondary: mortality |
| **Zullo et al, 2020 [16]#** | Observational study (retrospective cohort) | Institutionalized, >65 years, with osteoporosis /  United States | 6 years  Mean 2.6 | Scale generated in the Minimum Data Set (version 2.0), Online Survey Certification and Reporting System (variables derived frailty indices based on the accumulated deficits model) | NT: 23,101  NW: 19,476 (84.3%)  NI: 17,753  Mean age: 83.4 years (± 7.8) | Intervention:  Oral or intravenous bisphosphonate  Comparator:  Calcitonin | Primary: all fractures, hip fractures |
| **Zullo et al, 2019 [17]#** | Observational study (retrospective cohort) | Institutionalized,  >65 years, with osteoporosis /  United States | 6 years  mean 2.5  (± 7.8) | Scale generated in the Minimum Data Set (version 2.0), Online Survey Certification and Reporting System (variables derived frailty indices based on the accumulated deficits model) | NT: 24,571  NW: 20,956 (85.3%)  NI:5.209  Mean age: 83.4 years (± 7.8) | Intervention:  Oral or intravenous bisphosphonate  Comparator:  Calcitonin | Primary: hip fractures, non-vertebral fractures*; severe esophagitis ^1^  Secondary: mortality |
| **Sambrok et al, 2011 [27]** | Observational study (prospective cohort) | Institutionalized,  >65 years, with osteoporosis /  Australia | 5 years  Mean 3.4 | Any condition requiring nursing support or care | NT: 2,001  NW: 1,529 (76%)  NI:78  Mean age: 85.6 years (± 6.6) | Intervention: Oral bisphosphonate  Comparator:  no treatment | Primary: hip fractures* and mortality*  Secondary: cause-specific mortality |
| **Greenspan et al, 2015 [28]** | Randomized, placebo-controlled, double-blind clinical trial | Institutionalized>65 years, with BMD < −2.0 SD at the spine, hip or radius) or a prior vertebral or hip fracture/  United States | 2 years | Any condition requiring nursing support or care | NT: 181  NW:181 (100%)  NI: 89  Mean age:  85.4 years (± 0.6) | Intervention:  Single dose of zoledronic acid 5mg  Comparator:  Placebo | Primary: change in bone mineral density at the hip and spine  Secondary: adverse events, bone turnover markers, physical and cognitive function, all fractures, vertebral fractures, non-vertebral fractures*, mortality*,  change in BMD at other skeletal sites, comorbidity. |
| **Chattaris et al,**  **2022 [29]** | Observational study (retrospective cohort) | Medicare fee-for-service beneficiaries  ≥65 years, with previous fracture/  United States | 2 years | FI (fragility index by accumulated deficit) ≥ 0,2 (6 months before fracture) | NT: 29,904  NW: 22,131 (74%)  NF: 15,345  NI: 2,148  Mean age:  82 years (± 8.5) | Intervention:  - Oral or intravenous bisphosphonate  - Denosumab  - Teriparatide  Comparator:  no treatment | Primary: new fractures ≥ 100 days after the first  Secondary: mortality and re-hospitalization |

\# These articles are related to the same study. The review authors considered only exclusive data from each article.

1- Mortality data was presented but not indicated as an outcome in the study methods description.

NT: total number of participants; NW: number of women; NF: number of frail participants; NI: number of participants who received intervention; Mean age: from the intervention.

*These outcomes were included in the metanalysis

**Table S2.** Assessment of Risk of Bias in Observational Studies by ROBINS-I

| Study | Confounding | Intervention classification | Selection of participants | Intervention deviation | Missing data | Outcome measurement | Selective reporting | Overall risk |  |
| --- | --- | --- | --- | --- | --- | --- | --- | --- | --- |
| Zullo et al, 2021 [15] | Serious | Low | Low | Low | Low | Low | Low | Low* | |
| Zullo et al, 2020 [16] | Serious | Low | Low | Low** | Low | Low | Low | Low* | |
| Zullo et al, 2019 [17] | Serious | Low | Low | Low | Low | Low | Low | Low* | |
| Sambrook et al, 2011 [27] | Serious | Low | Low | Low | Low | Low | Low | Low* | |
| Chattaris et al, 2022 [28] | Moderate | Low | Moderate | Low | Low | Low | Low | Moderate | |

***Low risk of bias except for concerns about uncontrolled confounding.

**From a per-protocol perspective, this domain would have been classified as serious risk of bias.

**Table S3.** Assessment of the Risk of Bias in the Randomized Clinical Trial by RoB 2

| Study | Randomization | Intervention deviation | Missing data | Outcome measurement | Selective reporting | Overall risk |
| --- | --- | --- | --- | --- | --- | --- |
| Hosoi et al, 2025 [14] | Some concerns | Low** | High | Low | Some concerns | High |
| Greenspan et al, 2015 [28] | Low | Low | Low | Low | Some concerns | Some concerns |

**From a per-protocol perspective, this domain would have been classified as high risk of bias.

**SUMMARY OF THE SEARCH STRATEGIES**

| **Database** | **Records retrieved** |
| --- | --- |
| MEDLINE (via PubMed) | 79 |
| Cochrane Library (CENTRAL) | 23 |
| LILACS (via VHL) | 1 |
| Embase (via Elsevier) | 195 |
| Total records before deduplication | 298 |
| Duplicates removed (via Rayyan) | 60 |
| Total after deduplication | 238 |
| **Total number of studies included in the review** | 7 |

Duplicate management: screening and removal of duplicate records across the four databases were performed using Rayyan software, resulting in the removal of 60 duplicate records, leaving 238 unique records for title/abstract screening.

**1. MEDLINE (via PubMed)**

Search date: March 3, 2026
Coverage period: from database inception to March 3, 2026
Records retrieved: 79
Language restriction: none
Filters/limits applied: none
Search strategy: a combined strategy was used, applying the MeSH descriptor of the main term associated (OR) with free-text entry terms/synonyms, with no field restriction (all fields), with PubMed's Automatic Term Mapping feature active for free-text terms, without the use of field tags (e.g., [tiab]).

#1 "Aged"[Mesh] OR Elderly

#2 "Frailty"[Mesh] OR Frailties OR Frailness OR Frailty Syndrome OR Debility OR Debilities

#3 "Frail Elderly"[Mesh] OR Elderly, Frail OR Frail Elders OR Elder, Frail OR Elders, Frail OR Frail Elder OR Frail Older Adults OR Adult, Frail Older OR Adults, Frail Older OR Frail Older Adult OR Older Adult, Frail OR Older Adults, Frail OR Functionally-Impaired Elderly OR Elderly, Functionally-Impaired OR Functionally Impaired Elderly

#4 "Osteoporosis"[Mesh] OR Osteoporoses OR Osteoporosis, Age-Related OR Osteoporosis, Age Related OR Age-Related Osteoporosis OR Age-Related Osteoporoses OR Age Related Osteoporosis OR Osteoporoses, Age-Related OR Bone Loss, Age-Related OR Age-Related Bone Loss OR Age-Related Bone Losses OR Bone Loss, Age Related OR Bone Losses, Age-Related OR Osteoporosis, Senile OR Osteoporoses, Senile OR Senile Osteoporoses OR Senile Osteoporosis OR Osteoporosis, Involutional OR Osteoporosis, Post-Traumatic OR Osteoporosis, Post Traumatic OR Post-Traumatic Osteoporoses OR Post-Traumatic Osteoporosis

#5 "Osteoporosis, Postmenopausal"[Mesh] OR Osteoporosis, Post-Menopausal OR Osteoporoses, Post-Menopausal OR Osteoporosis, Post Menopausal OR Post-Menopausal Osteoporoses OR Post-Menopausal Osteoporosis OR Postmenopausal Osteoporosis OR Osteoporoses, Postmenopausal OR Postmenopausal Osteoporoses OR Perimenopausal Bone Loss OR Bone Loss, Postmenopausal OR Bone Losses, Postmenopausal OR Postmenopausal Bone Losses OR Postmenopausal Bone Loss OR Bone Loss, Perimenopausal OR Bone Losses, Perimenopausal OR Perimenopausal Bone Losses

#6 "Denosumab"[Mesh] OR AMG 162 OR Xgeva OR Prolia

#7 "Teriparatide"[Mesh] OR hPTH (1-34) OR Human Parathyroid Hormone (1-34) OR Parathar OR Teriparatide Acetate OR Forteo

#8 "Diphosphonates"[Mesh] OR Bisphosphonate OR Bisphosphonates

#9 "Aminohydroxybutane Bisphosphonate"[Mesh] OR 4-Amino-1-Hydroxybutylidene 1,1-Biphosphonate OR MK-217 OR MK217 OR MK 217 OR Fosamax OR Alendronate Sodium OR Alendronate Monosodium Salt, Trihydrate

#10 "Bisphosphonate Risedronate Sodium"[Mesh] OR Risedronate Sodium, Bisphosphonate OR Sodium, Bisphosphonate Risedronate OR Risedronate Sodium OR Actonel OR Risedronic Acid, Monosodium Salt OR Risedronate OR 1-Hydroxy-2-(3-pyridyl) ethylidene diphosphonate OR Atelvia OR 2-(3-pyridinyl)-1-hydroxyethylidene-bisphosphonate OR 2-(3-pyridinyl)-1-hydroxyethylidenebisphosphonate

#11 "2-(Imidazol-1-yl)-1-hydroxyethylidene-1,1-bisphosphonic acid"[Mesh] OR Zoledronic Acid Anhydrous OR CGP 42446A OR CGP 42'446 OR CGP-42'446 OR CGP42'446 OR CGP-42446 OR CGP42446 OR CGP 42446 OR Zometa OR Zoledronate

#12 "BA058"[Mesh] OR tymlos

#13 "AMG 785"[Mesh] OR AMG-785 OR AMG785 OR CDP 7851 OR CDP-7851 OR CDP7851 OR evenity OR romosozumab-aqqg

#14 #1 AND (#2 OR #3) AND (#4 OR #5) AND (#6 OR #7 OR #8 OR #9 OR #10 OR #11 OR #12 OR #13)

**2. Cochrane Library — CENTRAL**

Search date: March 3, 2026
Coverage period: from inception to March 3, 2026
Records retrieved: 23
Language restriction: none
Filters/limits applied: none
Search strategy: a pure free-text strategy was used, applying the main term and its respective entry terms/synonyms, all as free text (exact phrase), within the standard search fields (title, abstract, and keywords), without the use of a controlled descriptor (MeSH). Free-text searching was chosen for this database because a substantial proportion of CENTRAL records — originating from trial registries and conference abstracts — lack consistent MeSH indexing, which limits the yield of a descriptor-based search in this context.

#1 "Aged" OR Elderly

#2 "Frailty" OR Frailties OR Frailness OR Frailty Syndrome OR Debility OR Debilities

#3 "Frail Elderly" OR Elderly, Frail OR Frail Elders OR Elder, Frail OR Elders, Frail OR Frail Elder OR Frail Older Adults OR Adult, Frail Older OR Adults, Frail Older OR Frail Older Adult OR Older Adult, Frail OR Older Adults, Frail OR Functionally-Impaired Elderly OR Elderly, Functionally-Impaired OR Functionally Impaired Elderly

#4 "Osteoporosis" OR Osteoporoses OR Osteoporosis, Age-Related OR Osteoporosis, Age Related OR Age-Related Osteoporosis OR Age-Related Osteoporoses OR Age Related Osteoporosis OR Osteoporoses, Age-Related OR Bone Loss, Age-Related OR Age-Related Bone Loss OR Age-Related Bone Losses OR Bone Loss, Age Related OR Bone Losses, Age-Related OR Osteoporosis, Senile OR Osteoporoses, Senile OR Senile Osteoporoses OR Senile Osteoporosis OR Osteoporosis, Involutional OR Osteoporosis, Post-Traumatic OR Osteoporosis, Post Traumatic OR Post-Traumatic Osteoporoses OR Post-Traumatic Osteoporosis

#5 "Osteoporosis, Postmenopausal" OR Osteoporosis, Post-Menopausal OR Osteoporoses, Post-Menopausal OR Osteoporosis, Post Menopausal OR Post-Menopausal Osteoporoses OR Post-Menopausal Osteoporosis OR Postmenopausal Osteoporosis OR Osteoporoses, Postmenopausal OR Postmenopausal Osteoporoses OR Perimenopausal Bone Loss OR Bone Loss, Postmenopausal OR Bone Losses, Postmenopausal OR Postmenopausal Bone Losses OR Postmenopausal Bone Loss OR Bone Loss, Perimenopausal OR Bone Losses, Perimenopausal OR Perimenopausal Bone Losses

#6 "Denosumab" OR AMG 162 OR Xgeva OR Prolia

#7 "Teriparatide" OR hPTH (1-34) OR Human Parathyroid Hormone (1-34) OR Parathar OR Teriparatide Acetate OR Forteo

#8 "Diphosphonates" OR Bisphosphonate OR Bisphosphonates

#9 "Aminohydroxybutane Bisphosphonate" OR 4-Amino-1-Hydroxybutylidene 1,1-Biphosphonate OR MK-217 OR MK217 OR MK 217 OR Fosamax OR Alendronate Sodium OR Alendronate Monosodium Salt, Trihydrate

#10 "Bisphosphonate Risedronate Sodium" OR Risedronate Sodium, Bisphosphonate OR Sodium, Bisphosphonate Risedronate OR Risedronate Sodium OR Actonel OR Risedronic Acid, Monosodium Salt OR Risedronate OR 1-Hydroxy-2-(3-pyridyl)ethylidene diphosphonate OR Atelvia OR 2-(3-pyridinyl)-1-hydroxyethylidene-bisphosphonate OR 2-(3-pyridinyl)-1-hydroxyethylidenebisphosphonate

#11 "2-(Imidazol-1-yl)-1-hydroxyethylidene-1,1-bisphosphonic acid" OR Zoledronic Acid Anhydrous OR CGP 42446A OR CGP 42'446 OR CGP-42'446 OR CGP42'446 OR CGP-42446 OR CGP42446 OR CGP 42446 OR Zometa OR Zoledronate

#12 "BA058" OR tymlos

#13 "AMG 785" OR AMG-785 OR AMG785 OR CDP 7851 OR CDP-7851 OR CDP7851 OR evenity OR romosozumab-aqqg

#14 #1 AND (#2 OR #3) AND (#4 OR #5) AND (#6 OR #7 OR #8 OR #9 OR #10 OR #11 OR #12 OR #13)

**3. LILACS (via VHL — Virtual Health Library)**

Search date: March 3, 2026
Coverage period: from database inception to March 3, 2026
Records retrieved: 1
Language restriction: none
Filters/limits applied: none
Free-text field scope: free-text terms were searched restricted to the Title, Abstract, and Subject fields — the broadest option available in the VHL advanced search interface, which did not offer an "all indexes" option at the time of the search.
Symbol notation: the "$" symbol was used for truncation/explosion of DeCS descriptors, also retrieving hierarchically subordinate terms within the controlled vocabulary structure.
Additional methodological note: search blocks intentionally and redundantly combine the DeCS descriptor by name (MH:"term") and by its corresponding hierarchical numeric code (MH:code) for the same concept, as a safeguard to ensure descriptor retrieval within the VHL/LILACS interface.

#1 MH:"Idoso" OR (Aged) OR (Anciano) OR (Sujet âgé) OR MH:01.060.116.100

#2 MH:"Fragilidade" OR (Frailty) OR (Fragilidad) OR (Fragilité) OR MH:C23.550.359

#3 MH:"Idoso Fragilizado" OR (Frail Elderly) OR (Anciano Frágil) OR (Personne âgée fragile) OR MH:M01.060.116.100.540

#4 MH:"Osteoporose" OR (Osteoporosis) OR (Ostéoporose) OR MH:C05.116.198.579$ OR MH:C18.452.104.579$

#5 MH:"Osteoporose Pós-Menopausa" OR (Osteoporosis, Postmenopausal) OR (Osteoporosis Posmenopáusica) OR (Ostéoporose post-ménopausique) OR MH:C05.116.198.579.610$ OR MH:C18.452.104.579.610$

#6 MH:"Denosumab" OR (Dénosumab) OR MH:D12.776.124.486.485.114.224.060.782$ OR MH:D12.776.124.790.651.114.224.060.782$ OR MH:D12.776.377.715.548.114.224.200.782$

#7 MH:"Teriparatida" OR (Teriparatide) OR (Teriparatido) OR (Tériparatide) OR MH:D06.472.699.590.850$ OR MH:D12.644.548.587.850$

#8 MH:"Difosfonatos" OR (Diphosphonates) OR MH:D02.705.429.500$

#9 MH:"Alendronato" OR (Alendronate) OR (Alendronato) OR (Alendronate) OR MH:D02.705.429.500.100

#10 MH:"Ácido Risedrônico" OR (Risedronic Acid) OR (Ácido Risedrónico) OR (Acide risédronique) OR MH:D02.705.429.500.872 OR MH:D03.383.725.836

#11 MH:"Ácido Zoledrônico" OR (Zoledronic Acid) OR (Ácido Zoledrónico) OR (Acide zolédronique) OR MH:D02.705.429.500.942 OR MH:D03.383.129.308.990

#12 MH:"abaloparatida" OR (abaloparatide)

#13 MH:"romosozumabe" OR (romosozumab)

#14 #1 AND (#2 OR #3) AND (#4 OR #5) AND (#6 OR #7 OR #8 OR #9 OR #10 OR #11 OR #12 OR #13)

**4. Embase (via Elsevier — Embase.com)**

Search date: March 3, 2026
Coverage period: from database inception to March 3, 2026
Records retrieved: 195
Language restriction: none
Filters/limits applied: none
Search strategy: a pure free-text strategy was used, applying the main term and its respective entry terms/synonyms, without the use of the database’s-controlled vocabulary (Entree).

#1 (Aged) OR (Elderly)

#2 (Frailty) OR (Frailties) OR (Frailness) OR (Frailty Syndrome) OR (Debility) OR (Debilities)

#3 (Frail Elderly) OR (Elderly, Frail) OR (Frail Elders) OR (Elder, Frail) OR (Elders, Frail) OR (Frail Elder) OR (Frail Older Adults) OR (Adult, Frail Older) OR (Adults, Frail Older) OR (Frail Older Adult) OR (Older Adult, Frail) OR (Older Adults, Frail) OR (Functionally-Impaired Elderly) OR (Elderly, Functionally-Impaired) OR (Functionally Impaired Elderly)

#4 (Osteoporosis) OR (Osteoporoses) OR (Osteoporosis, Age-Related) OR (Osteoporosis, Age Related) OR (Age-Related Osteoporosis) OR (Age-Related Osteoporoses) OR (Age Related Osteoporosis) OR (Osteoporoses, Age-Related) OR (Bone Loss, Age-Related) OR (Age-Related Bone Loss) OR (Age-Related Bone Losses) OR (Bone Loss, Age Related) OR (Bone Losses, Age-Related) OR (Osteoporosis, Senile) OR (Osteoporoses, Senile) OR (Senile Osteoporoses) OR (Senile Osteoporosis) OR (Osteoporosis, Involutional) OR (Osteoporosis, Post-Traumatic) OR (Osteoporosis, Post Traumatic) OR (Post-Traumatic Osteoporoses) OR (Post-Traumatic Osteoporosis)

#5 (Osteoporosis, Postmenopausal) OR (Osteoporosis, Post-Menopausal) OR (Osteoporoses, Post-Menopausal) OR (Osteoporosis, Post Menopausal) OR (Post-Menopausal Osteoporoses) OR (Post-Menopausal Osteoporosis) OR (Postmenopausal Osteoporosis) OR (Osteoporoses, Postmenopausal) OR (Postmenopausal Osteoporoses) OR (Perimenopausal Bone Loss) OR (Bone Loss, Postmenopausal) OR (Bone Losses, Postmenopausal) OR (Postmenopausal Bone Losses) OR (Postmenopausal Bone Loss) OR (Bone Loss, Perimenopausal) OR (Bone Losses, Perimenopausal) OR (Perimenopausal Bone Losses)

#6 (Denosumab) OR (AMG 162) OR (Xgeva) OR (Prolia)

#7 (Teriparatide) OR "hPTH (1-34)" OR "Human Parathyroid Hormone (1-34)" OR (Parathar) OR (Teriparatide Acetate) OR (Forteo)

#8 (Diphosphonates) OR (Bisphosphonate) OR (Bisphosphonates)

#9 (Aminohydroxybutane Bisphosphonate) OR (4-Amino-1-Hydroxybutylidene 1,1-Biphosphonate) OR (MK-217) OR (MK217) OR (MK 217) OR (Fosamax) OR (Alendronate Sodium) OR (Alendronate Monosodium Salt, Trihydrate)

#10 (Bisphosphonate Risedronate Sodium) OR (Risedronate Sodium, Bisphosphonate) OR (Sodium, Bisphosphonate Risedronate) OR (Risedronate Sodium) OR (Actonel) OR (Risedronic Acid, Monosodium Salt) OR (Risedronate) OR (1-Hydroxy-2-(3-pyridyl)ethylidene diphosphonate) OR (Atelvia) OR (2-(3-pyridinyl)-1-hydroxyethylidene-bisphosphonate) OR (2-(3-pyridinyl)-1-hydroxyethylidenebisphosphonate)

#11 (2-(Imidazol-1-yl)-1-hydroxyethylidene-1,1-bisphosphonic acid) OR (Zoledronic Acid Anhydrous) OR (CGP 42446A) OR (CGP 42'446) OR (CGP-42'446) OR (CGP42'446) OR (CGP-42446) OR (CGP42446) OR (CGP 42446) OR (Zometa) OR (Zoledronate)

#12 (BA058) OR (tymlos)

#13 (AMG 785) OR (AMG-785) OR (AMG785) OR (CDP 7851) OR (CDP-7851) OR (CDP7851) OR (evenity) OR (romosozumab-aqqg)

#14 #1 AND (#2 OR #3) AND (#4 OR #5) AND (#6 OR #7 OR #8 OR #9 OR #10 OR #11 OR #12 OR #13)
